# Resting disparity in quoll semelparity: examining the sex-linked behaviours of wild roaming northern quolls (*Dasyurus hallucatus*) during breeding season

**DOI:** 10.1098/rsos.221180

**Published:** 2023-02-01

**Authors:** Joshua L. Gaschk, Kaylah Del Simone, Robbie S. Wilson, Christofer J. Clemente

**Affiliations:** ^1^ School of Science, Technology and Engineering, University of the Sunshine Coast, Sippy Downs, QLD 4556, Australia; ^2^ School of Biological Sciences, University of Queensland, St Lucia, QLD 4067, Australia

**Keywords:** behaviour, Dasyuridae, activity budgets, accelerometers, semelparity, sleep deprivation

## Abstract

Semelparity is a breeding strategy whereby an individual invests large amounts of resources into a single breeding season, leading to the death of the individual. Male northern quolls (*Dasyurus hallucatus*) are the largest known mammal to experience a post-breeding die-off; however, the cause of their death is unknown, dissimilar from causes in other semelparous dasyurids. To identify potential differences between male northern quolls that breed once, and females that can breed for up to four seasons, the behaviours, activity budgets, speeds and distances travelled were examined. Northern quolls were captured on Groote Eylandt off the coast of the Northern Territory, Australia, and were fitted with accelerometers. A machine learning algorithm (Self-organizing Map) was trained on more than 76 h of recorded footage of quoll behaviours and used to predict behaviours in 42 days of data from wild roaming quolls (7M : 6F). Male northern quolls were more active (male 1.27 g*,* s.d. = 0.41; female 1.18 g, s.d. = 0.36), spent more time walking (13.09% male: 8.93% female) and engaged in less lying/resting behaviour than female northern quolls (7.67% male: 23.65% female). Reduced resting behaviour among males could explain the post-breeding death as the deterioration in appearance reflects that reported for sleep-deprived rodents.

## 1. Introduction

Semelparity is a reproductive strategy that is characterized by the die-off of one or both sexes after a single reproductive episode or season [1]. While a common strategy in long-lived individuals is to invest in multiple episodes of breeding (iteroparity), some short-lived species maximize investment during their first breeding season to such an extent that their chances of surviving to a second breeding season are greatly reduced (semelparity) [2]. All instances of semelparity in mammals occur in the marsupial families Dasyuridae (19 species) and Didelphidae (3 species) [3–5]. Most marsupials that engage in a semelparous lifestyle seem to live in locations with a seasonally predictable increase of prey abundance coinciding with breeding and weaning [6]. Females of semelparous species have synchronized and reduced the length of their breeding season, increasing sexual selection and sperm competition which results in the males increasing their mating effort [7,8].

The male die-off in most semelparous dasyurids is identified by a post-breeding lethargic/timid demeanour, weight loss, general degradation of health (fur & weight loss, parasite infestation), the degeneration of testes and spermatogenic failure [6,9–11]. The cause of this die-off has been studied extensively in smaller species of Dasyuridae [9,12–16]. The loss of condition in males was closely examined in the Brown antechinus (*Antechinus stuartii*) and the red-tailed phascogale (*Phascogale calura*), showing a correlative link with an increased production of corticosteroids [17,18]. Females displayed an increased ability to bind corticosteroids using corticosteroid-binding globulins; however, their action in males is reduced. The excess corticosteroids are suggested to cause stomach ulcers, gastrointestinal haemorrhaging and liver abscesses, which become fatal [10,18]. When forced to abstain from breeding, male antechinus did not produce the excessive amounts of corticosteroid and survived until they were allowed to breed in a subsequent season [19]. Yet this change in the endocrine system reported in small dasyurids has not been observed in the largest semelparous marsupial, the northern quoll (*Dasyurus hallucatus*) [10]. Corticosteroid production does not increase in male northern quolls and at time of death they do not have the internal complications present in antechinus and phascogales [10]. It is currently unknown what causes the die-off in male northern quolls with the only possible suggestion a type of post-breeding senescence [20]. Behavioural choices made by males could reveal the causal factors behind the die-off; however, this can be difficult to obtain in a cryptic species like northern quolls.

Accelerometer technology in combination with machine learning is a novel approach to understanding the daily behaviours of animals, particularly those that are difficult to observe naturally [21,22]. To date, they have been commonly used to determine energy expenditure and broad-scale behaviours (e.g. locomotion/stationary-inactive/stationary-active) [23]. These devices have shown increasing success in determining fine-scale behaviours in domestic cats and dingoes [21,24,25]. Thus, differences between fine-scale behavioural repertoires and activity budgets of male and female northern quolls might be linked with this species’ breeding strategy.

Reports that male quolls forgo basic survivability, travelling large distances to maximize breeding partners, would suggest that they would be much more active than their female counterparts [26,27]. We employed accelerometer technology to explore these potential behavioural changes between males and females. If males are more active than female quolls, this difference should be evident from differences in activity budgets. This difference might also be represented in the timing of specific behaviours. We expect males to use locomotor behaviours (walking etc.) for longer periods of time than females, to support suggestions that they travel great distances for the purpose of mating. The alternative hypothesis is that there is no difference in activity or behavioural repertoires and the die-off that males experience is due to an unexplored aspect of their physiology.

## 2. Materials and methods

### 2.1. Quoll capture and device attachment

Northern quolls were trapped on Groote Eylandt during breeding season July–August 2019, indicated by the breeding scars on the females resulting from the copulatory process. Trapping was conducted at three sites, one was a rocky outcrop with increased variation in topography (−13.891193, 136.531090), a eucalypt woodland with dense undergrowth (−13.969006, 136.472657) and mixed habitat near urban living (−13.842370, 136.416434). Tomahawk original series cage traps (14 × 14 × 45 cm; Tomahawk ID-101SS, Hazelhurst, Wisconsin, USA) were set up in grids, four rows (100 m apart) of 10 traps (60 m apart) and baited with canned dog food. Traps were checked every morning and captured quolls were placed in individual calico bags (550 × 750 mm; Westernex SKU : B53, Brisbane, Australia) and transported to the Anindilyakwa Land and Sea Ranger Research Station for processing and accelerometer attachment. Each quoll was microchipped between the shoulder blades (Trovan nanotransponder ID-100, Keysborough, VIC, Australia) and marked with an ear tag (1005–1 MONEL, National Band and Tag Co.) for individual identification. Processing of individual quolls followed methods previously recorded in [28]. All research methodologies were approved by the University of Queensland's Animal Ethics Committee (permit number SBS/085/19) and the Northern Territory Parks and Wildlife Commission (permit number 58566).

### 2.2. Device attachment

Northern quolls that were more than 280 g (such that the device and harness was less than 5% of body mass) had accelerometers (Axivity AX3, manufacturer, 23 × 32.5 × 7.6 mm; 11 g) fitted using a custom-designed felt harness. A piece of felt, doubled over for thickness, was folded into a ‘V’ shape, with a base of approximately 3 cm in length for the device. The accelerometer was glued and shrink-wrapped to the base and any sharp edges of the shrink-wrap were removed using scissors and a file. The device and strap were secured onto the back of the animal, above the shoulder blades, so that the felt was between the device and the animal. The device was orientated so that X + was posterior, Y + was right lateral and Z + was ventral and devices set to record at 50 Hz with a g-range of ±8 g. The extremities of the straps were wrapped toward the front of the animal, over the shoulder, under the chest and across the body and joined with the diagonally opposite side of the device using cable ties. The device was attached so that two fingers would fit under the straps to avoid unnecessary discomfort to the animal and ensure that the animal could move appropriately. Excess felt was removed from the harness to limit weight and the harness checked to ensure that no limbs were caught in the straps and that the animal could move freely. The harness was weighed before attachment to ascertain the weight and guarantee that it did not exceed the 5% threshold.

### 2.3. Collection of training data

Following attachment, quolls were placed in two enclosures to collect the behaviour training data for the prediction algorithm. The first was a 0.6 m wide ‘L-shaped’ trackway that extended for 3 m in one direction and 1.5 in the perpendicular direction. Walls of this enclosure were 1.5 m tall plyboard and lined with corflute to prevent escape. The floor was covered in foam mats to protect the quoll during jumps. The second enclosure was a 1.5 × 1 × 0.2 m box with a clear acrylic sheet on one side and open at the top. Animals were left to behave as naturally as possible for 5-10 min in either enclosure to adjust to the harness. After this time period either the animal was encouraged to run (in the L-shaped enclosure) or climb on a piece of dowel covered in sandpaper (2 cm in diameter), for 2-5 min. After filming the animals, the training data was removed from the accelerometer and devices were reset. All actions were filmed with a high-speed digital camera (240 fps: Casio EXILIM HS EX-ZR200). To synchronize the clock between the accelerometer and the cameras, each device was tapped five–six times, while in view of one of the cameras before the device was attached.

Animals were then released at the place of capture with the backpacks attached. The harness was designed to degrade and fall off the animal on its own accord after 2–9 days. After recapture, if the device was present, the data were removed, the device reset, and the animal was weighed. If the animal was still in healthy condition (no significant weight loss, hair loss, etc. and device still tight), the animal was released with the device again. If there was significant weight and condition loss (in males), the device was removed. This resulted in an approximate total of 850 h (mean = 70.9, s.e. = 56.25) of *in situ* data across 13 northern quolls (6 female: 7 male).

### 2.4. Behaviour identification and video tagging

Accelerometer traces (XYZ force data; training and wild roaming) were exported from the devices as raw CSV files using the OMGUI software package (V1.0.0.43, Axivity). Videos with specific activities were split from the original videos into 10–60 s chunks. The behaviours recorded during the training data collection were Climbing, Resting/Lying, Sitting, Vigilance, Standing Vigilance, Vigilant Walking, Walking, Turning, Galloping, Bounding, Jumping and Scurrying (electronic supplementary material, table S1). Lying/Resting data were collected from within these enclosures but also when the animals were resting in their individual calico bags in air-conditioned facilities before release in the late afternoon. General behaviours, such as eating, drinking and any intraspecific interactions, were unattainable due the unfamiliarity of the laboratory conditions discouraging these behaviours and the restrictions for placing multiple northern quolls in the same enclosures.

### 2.5. Data processing and analysis

The video alignment, behavioural identification, data processing, Self-organizing Map (SOM) creation and behaviour prediction follow the methods from Galea *et al.* [21], and only a brief description is given here (see electronic supplementary material, Appendix 1). SOMs are artificial neural networks that are often used for extraction and prediction of large datasets by identifying patterns in multi-dimensional data. The SOM creation process involved a large dataset of 1 s epochs associated with a number of behaviours (12) that are identified by predictor variables (25) and splitting this dataset into training data (80%) and testing (20%). These predictor variables were chosen based on a sensitivity analysis described in Tatler, Cassey [24], and were means, s.e., signal magnitude area, overall body accelerations (OBA), vectorial body accelerations (VBAs), skews, and axes correlations and are described further in the electronic supplementary material, table S2. The dataset used for this training/testing process contained 2 74 695, 1 s observations of our 25 predictors (approx. equal to 76 h) of 19 northern quolls (8F : 11M) and an SOM model with the grid shape of 7 by 10 was created.

A model for all behaviours was created from the training dataset using an SOM with the package kohonen in Rstudio (Ver. 3.0.10) [29,30]. The accuracy of the SOM prediction model was tested using a confusion matrix, extracting the standard accuracy measures, specificity, precision, sensitivity and overall accuracy (electronic supplementary material, table S3). The ‘predict‘ function from the kohonen package used the SOM algorithm to predict the behaviours in the wild roaming northern quolls.

### 2.6. Vectorial body acceleration

VBA was employed as a proxy for energy use in northern quolls and to the compare the energy use in specific behaviours [31]. It differs from dynamic body acceleration (DBA) as it includes the static acceleration from gravity that acts on the device. As well as a proxy for energy use it was used as a predictor variable in the creation of the SOM, and to predict speed and distance travelled in wild roaming northern quolls. VBA was chosen instead of OBA because of its capacity to account for directional changes in the accelerometer device [31]. The mean maximum VBA_max_ (maximum over a window of 1 s) was aggregated across hour and day in all northern quolls to determine how they expend energy throughout the day.

To use VBA to predict speed, videos of northern quolls locomoting in the trackway were analysed using Dltdv (Ver 7) [32]. Distance was calibrated based on known markings on the floor, and the frame rate of the videos was used to calculate duration. Strides within the videos were tagged or marked (placing a virtual marker in the video frame) at the touchdown of either of the hind feet to subsequent touch downs of the same hindfoot, incorporating all footfalls. Speed was calculated by tagging or marking either the head or tail-base of the animal in consecutive frames of the video. These bouts were then identified within the training accelerometer traces and extracted to produce an average VBA over the duration of the bout. A linear model was created using Log_10_ transformed Average VBA (g) of each individual bout as the predictor variable and Log_10_ Speed (m s^−1^) as the response variable. Log transformation of data was necessary to normalize residuals.

The bouts of continuous locomotor behaviours from wild roaming quolls were extracted from the SOM behaviour predictions. The average VBA was then calculated over the duration of the locomotor bouts and Log_10_ transformed to predict the average speed. Average VBA was not used to create the behavioural prediction algorithm (SOM), and the behaviours were predicted independently of this variable. The average speed was then applied to the duration of the bout to get a predicted distance travelled. DBA has been shown to correlate with distance travelled in dingoes and with speed in humans [25,33]. This process resulted in 24 374 and 48 344 estimated locomotor bouts for females and males, respectively. Gallops were not able to be identified during video analysis. To predict this behaviour in wild roaming quolls, we classified gallops as walking gaits; walks (not trots or paces) have a similar beat pattern to gallops (3–4 beats) as opposed to bounds which possess a two-beat gait pattern [34].

Continuous bouts of stationary behaviours (Lying/Resting and Sitting) were also extracted from the SOM behaviour predictions. Length of stationary bouts was converted to minutes. This process resulted in 2918 female and 5152 male total stationary bouts where individuals assumed the lying/resting or sitting position. To distinguish between a pause in activity and rest that would likely result in sleep, bouts of less than 10 min removed from the dataset, based on the mean sleep cycle length 22.5 min (s.e. = 1.9) in common opossums (*Didelphis marsupialis*) [35]. This process resulted in 278 female and 384 male bouts of longer than 10 min. Analyses were performed on both datasets, firstly linear mixed effects models on the duration of sleep, and a permutation test of equality between two densities. The permutation test of equality was calculated using the function sm.density.compare from the package ‘sm’ (Version 2.2-5.7) [36].

### 2.7. Statistical analysis

Sex linked behavioural variation was analysed using linear mixed effects models (lme) from the ‘nlme’ package in R (Ver. 1.7.4-1; Models 3, 5-10) [37]. Full models included subject nested in hour (hour of the day 0 : 23) and days (date of collection) as a random effect. Models were simplified using Akaike Information Criterion (AIC) scores by comparing two models and choosing the model with the lowest AIC score. Hour was considered a fixed effect when we were testing explicitly for its influence on dependent variables (i.e. activity or behaviour in quolls throughout the day, Models 3 and 5). When Count (the number of times one second of behaviour was used in an hour) was the explanatory variable, models were calculated using a Poisson distribution with subject as a random effect (Models 1, 2 and 4). This was calculated using the function glmer from the package ‘lme4’ and *p*-values were calculated using the function pf from the ‘stats’ package in base R [38]. Tukey *post hoc* tests were computed using the ‘emmeans’ package in R [39]. More details of linear models and *post hoc* tests can be found in the electronic supplementary material, tables S5 and S6.

## 3. Results

### 3.1. Behaviour prediction

All 12 behaviours were predicted with a sensitivity ≥ 98.10%, precision ≥ 97.86%, specificity ≥ 99.94% and overall accuracy ≥ 99.94% (electronic supplementary material, table S4). Scurries and climbing behaviours were not predicted in either male or female wild roaming quolls despite being predicted accurately in the training data (electronic supplementary material, figure S1*a*).

**Figure 1.**
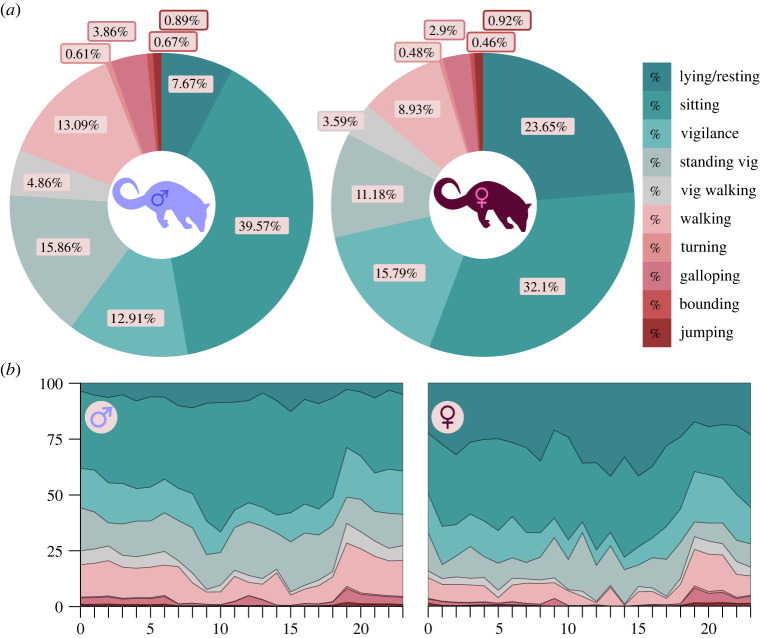
The behavioural breakdown between male (blue) and female (maroon) northern quolls based on the predictions from the 7 by 10 SOM algorithm. (*a*) The donuts represent the behavioural repertoires of northern quolls and percentage of total time spent in individual behaviours. (*b*) The area charts represent the percentage (%) of behaviours used during each hour of the day (24 h).

All 10 other behaviours were predicted in both sexes over 26.29 days of wild roaming males and 17.25 days of wild roaming females. Females spent 82.72% of their time in behaviours with low VBA_max_ (figure 1*a*; lying/resting, sitting, vigilance and standing vigilance). Male quolls spent 76.01% of their time in low-energy behaviours, less than that of females; however, this was insignificant (*f*_1,22_ = 2.8, *p* = 0.05, Model 1). When comparing the difference between specific low energetic behaviours, lying/resting, vigilance and standing vigilance showed a significant change between male (LR—7.67%, Vig—12.91%, SV—15.86%) and female quolls (LR— 23.65%, Vig—15.79%, SV—11.18%; *t*_1,22_ = 14.21, *p* < 0.001). Male and female northern quolls also showed a difference in their medium and high energetic behaviours. Females spent 12.52% and 4.76% using medium- and high-energy behaviours, respectively, and males 17.95% and 6.03%, respectively, with a significant difference between medium energetic behaviours (*z* = −3.60, *p* = 0.004). Males spent more time locomoting than females, walking 13.09% compared to 8.93%, Vig walking 4.86–3.59%, galloping 3.82–2.9% and bounding 0.6–0.46% (figure 1*a*; *f*_1,16423_ = 7.72 × 10^5^, Model 2); however, only walking was significant (*z* = −4.65, *p* < 0.001).

Hour of the day had no significant effects on the number of epochs recorded in wild roaming quolls, meaning there was a uniform sampling of epochs across hours throughout the days (*f*_1,23_ = 0.914, *p* = 0.5793). Behaviour (*f*_9, 27716_ = 1092.06, *p* < 0.001, Model 3), the behaviour : hour interaction (*f*_207, 27716_ = 4.55, *p* < 0.001, Model 3) and sex : behaviour interaction (*f*_9,14957_ = 85.65, *p* < 0.001) were all significant in predicted behaviours. This suggests that there was a difference in how quolls used their behaviours throughout the day. Figure 1*b* illustrates this shift, with a spike in medium- and high-energy behaviours between 19 : 00 and midnight (00 : 00 h). When performing a Tukey *post hoc* test on the difference in behaviour and hour between the sexes only lying/resting at 02 : 00 (*p* = 0.03), 12 : 00, (*p* = 0.04), 13 : 00 (*p* = 0.01) and 16 : 00 (*p* = 0.029; electronic supplementary material, table S6) were significantly different. Males rested much less than females in these four hours (figure 1*b*).

### 3.2. Behaviour energetics, activity budgets

The VBA_max_ of all behaviours were significantly different from one another except for low-energy use behaviours vigilance (1.26 g, s.e. = 0.29) and standing vigilance (1.24 g, s.e. = 0.30; *p* = 0.984), and sitting (1.04 g, s.e. = 0.13) and lying/resting (1.09 g*,* s.e. = 0.16; *p* = 0.208) (electronic supplementary material, figure S1*b*, Model 4). Bounding was the most energetically costly behaviour (6.76 g ± 1.74) and was significantly different from other high-energy behaviours jumping (5.91 g, s.e. = 1.29; *p* < 0.001), turning (3.71 g, s.e. = 0.76; *p* < 0.001) and galloping (3.61 g, s.e. = 1.16; *p* < 0.001). Energetically inexpensive locomotor behaviours, walking (1.96 g, s.e. = 0.81; *p* < 0.001) and vigilant walking (1.44 g, s.e. = 0.29; *p* < 0.001) differed significantly from the energetically costly locomotor forms gallops and bounds.

Sex of northern quolls had no influence on the VBA_max_ of different behaviours (*f*_9,1_ = 0.106, *p* = 0.751). Sex and time of day did determine total activity and when northern quolls were active; however, the interaction between the two was not significant (*f*_23,448_ = 3.42, *p* = 0.09). Over the duration of the study males had an average VBA_max_ of 1.27 g (s.e. = 0.38, *n* = 631) and females 1.18 g (s.e. = 0.33, *n* = 414). Both male and female northern quolls had low VBA_max_ between the hours of 7 : 00 and 18 : 00 (M—1.02 g, s.e. = 0.23; F—1.02 g, s.e. = 0.15). After this period, both males and females had their highest bout of activity from 19 : 00 which slowly declined until about 1 : 00 (19 : 00–01 : 00; M—1.40 g, s.e. = 0.40; F—1.31 g, s.e. = 0.43). Between the hours of 01 : 00 and 07 : 00 (M—1.12 g, s.e. = 0.42; F—1.06 g, s.e. = 0.28), male quolls seem to keep a constant medium level of activity while female activity dropped to levels just above resting similar to that observed in the middle of the day, with the exception of 04 : 00.

### 3.3. Predicted speeds

**Figure 2.**
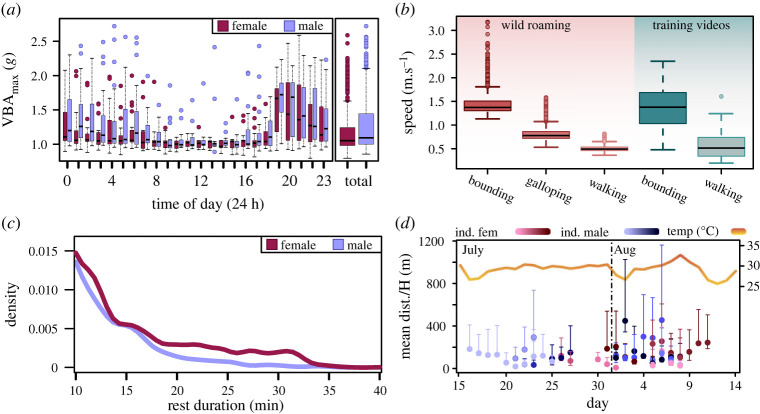
Examining four different aspects of behaviour using accelerometer technology, daily activity budgets, speed of locomotion, sleep duration and distance travelled per hour. (*a*) A boxplot of the maximum VBA of male (*n* = 7: blue) and female (*n* = 6: maroon) northern quolls across the hours of the day and total. The mean is represented by the thick black lines; the box includes the interquartile range. The hours represent the time between the start of that hour and the subsequent hour. (*b*) The predicted speeds (m s^−1^) of different locomotor gaits identified using the SOM in wild roaming northern quolls, compared to the speeds calculated from video analysis in the training videos. (*c*) The difference between male and females in frequency of uninterrupted resting duration greater than 10 min (min) in wild roaming male (blue) and female (maroon) northern quolls. (*d*) The mean distance travelled per hour (m) and the interquartile range (bars) across individual quolls and days (males—blue; females—maroon). The temperature (orange/yellow) shows the temperature (°C) range during the collection of data.

A mixed effects linear model was used to predict the speed and distance travelled in wild roaming northern quolls (electronic supplementary material, figure S2*a* and table S5). The interaction term between Log_10_ average VBA and behaviour showed a significant effect on the Log_10_ speed with a conditional *R*^2^ value of 85.3% (*F*_1,205_ = 61.47, *p* < 0.0001, Model 6). The mean speed of bounds (1.39 m s^−1^, s.e. = 0.45) and walks (0.56 m s^−1^, s.e. = 0.24) from the training videos were significantly different (figure 2*b*; *F*_1,4_ = 63 844.7, *p* < 0.0001; *t*_72913_ = 64.73, *p* < 0.001). All three of the mean speeds predicted for locomotor behaviours in wild roaming quolls were also significantly different from each other (BG—t_72913_ = 253.3, *p* < 0.001; BW—*t*_72913_ = 390.3, *p* < 0.001; GW—*t*_72913_ = 336.2, *p* < 0.001). Overall, wild roaming quolls spent on average 1.16 h per day walking at an average pace of 0.5 m s^−1^ (s.e. = 0.06), 8.39 min galloping at an average of 0.81 m s^−1^ (s.e. = 0.14), and 1 min bounding at 1.45 m s^−1^ (s.e. = 0.24). These percentages differed slightly from those predicted through the SOM because this analysis combined continuous bouts of locomotion. There was no significant difference between bounds calculated from videos and bounds predicted from the SOM (*t*_72913_ = 1.66, *p* = 0.458). There was, however, a significant difference in the means between wild roaming and video-calculated walking (figure 2*b*; *t*_72913_ = −5.81, *p* < 0.001). This is likely due to an inability to accurately identify galloping gaits in the video analysis. There was no difference between the speeds used by male and female wild roaming quolls (*F*_1,11_ = 2.05, *p* = 0.18, Model 7).

### 3.4. Distance travelled

Distance travelled for each individual bout in wild roaming northern quolls was calculated by taking the average speed of the bout and multiplying it by the bout's duration. The mean distance moved per day was estimated to be 3.13 km (s.e. = 2.23) for wild roaming northern quolls. While there was a significant difference between the distance travelled when using specific behaviours (*F*_1,71930_ = 15.37, *p* < 0.0001, Model 8), there was no significant difference between the distance travelled by males and females (electronic supplementary material, figure S2*b*; *F*_1,345_ = 0.001, *p* = 0.973). When examining this on an individual scale, there was also no significant difference in how individuals moved compared to each other (figure 2*d*; *F*_1,12_ = 0.55, *p* = 0.8659).

### 3.5. Stationary bouts

Continuous stationary bouts were extracted to determine if shorter rest duration contributed to the behavioural difference between male and female resting. Sex had a significant effect on how long males and females engaged in resting bouts for both total bouts (electronic supplementary material, figure S3*a* and table S5; *F*_1,262_ = 15.26, *p* < 0.001, Model 10) and also bouts for longer than 10 min (figure 2*c*; electronic supplementary material, table S5; F_1,179_ = 15.39, *p* < 0.001, Model 9). Females rested on average for 3.85 min (s.e. = 5.48) and males 3.12 min (s.e. = 3.82), and for stationary bouts greater than 10 min, females showed an average of 17.64 min (s.e. = 7.22) and males averaged 14.75 min (s.e. = 4.52). The density plots between male and female rest frequency showed no difference for all stationary bouts (*p* = 0.18); however, for stationary bouts for longer than 10 min the densities were dissimilar (*p* < 0.001; figure 2*c*).

## 4. Discussion

To understand the cause of die-off in male northern quolls, we explored the behavioural repertoire, temporal activity and distance travelled using accelerometers. This provided a means to explore the behaviours involved during the breeding season of northern quolls. The use of accelerometers to understand fine-scale behaviours is relatively novel in terrestrial landscapes, thus we first define general patterns of behaviour in northern quolls.

### 4.1. Accelerometers in animal behaviour

The time of activity in northern quolls was consistent with a nocturnal animal, with both males and females having their highest moments of activity between the hours of 19 : 00 and 00 : 00 and then slowly decreasing to sunrise (figure 2*a*). This pattern was different to temporal activity in eastern quolls (*D. viverrinus*), estimated from VHF tracking, which were reported to be active for 60% or more of observations throughout the night [40]. Even during the hours of peak activity, wild roaming northern quolls only spent approximately 30–35% in active behaviours (medium to high energy). The difference between these two activity measures could be related to the nature of direct observation of wild animals. Specific behaviours that are directly observable are more commonly detected outside of refuges; animals will have a purpose for roaming (hunting, social interaction etc.) and therefore are more likely to be active when observed (i.e. a bias towards active observable behaviour). An example of observable behaviour bias is evident in a study of yellow-bellied gliders by methods of spotlighting where they reported foraging behaviours (feeding, climbing and gliding) for 89.7%, and resting for only 1.8% of the time they were observed [41].

Northern quolls engaged in lying/resting behaviour throughout the night (figure 1*b*), similar to other species observed using accelerometer-based methods. For example, accelerometer-based observations showed two species of diurnal chipmunk (*Tamias alpinus*, *T. speciosus*) engaged in 20% or more of stationary or still behaviours throughout the day with the two other behaviours, movement and locomotion, recorded during the night [23]. Similarly, this variable pattern in behaviours was evident in the northern quolls, which suggested some locomotor behaviour still occurs throughout the day (nocturnal species); however, this could be skewed by the breeding season (figure 1*b*).

### 4.2. Bottom-up temporal activity

The temporal activity patterns in northern quolls were dissimilar to the patterns of spotted-tail quolls (*D. maculatus*), in Tasmania observed via camera traps. Spotted-tail quoll activity was reported to be dependent on the activities of apex predators, Tasmanian devils; their activity was bimodally distributed, with peaks at dusk and dawn when devils were absent, but showed only one peak before dawn if devils were in high abundance [42]. This pattern of temporal avoidance is a commonly supported theory for many non-apex predators, but it does not seem to be the reason behind temporal activity of the northern quolls on Groote Eylandt [43–46]. Dingoes, being the only mammalian, terrestrial predator on Groote Eylandt, operate around dusk and after dawn in open woodlands and pastures, increasing their activity between dusk and midnight in more complex habitats [47]. If the activity levels of dingoes on Groote Eylandt resemble those from central Queensland and Western Australia, it is not deterring temporal activity in northern quolls on Groote Eylandt, which remain active during dusk and dawn (figure 2*a*). The lack of behavioural modification in response to the presence of dingos might also explain why northern quoll populations are often negatively influenced by the presence of predators [27,48].

Instead, the spike in northern quoll activity (19 : 00; figure 2*a*) seems to be consistent with other small insectivorous dasyurids. The peak of activity at sunset was similar to observed activity in kowaris (*Dasyuroides byrnie*) and stripe-faced dunnarts (*Sminthopsis macroura*), where they were most frequently observed just after sunset with reduced observations throughout the night [49]. This peak in activity in northern quolls could then similarly be explained by the increased abundance of arthropod prey soon after sunset [50,51]. The reliance of their temporal activity on prey abundance has been reported previously; however, these studies found that northern quolls showed bimodal patterns which were dissimilar to quolls during breeding season [48,52,53]. The prolonged activity throughout the night, found in breeding quolls, could be explained by the additional requirement to be active during breeding.

### 4.3. Comparison of the sexes' locomotion

Male and female quolls differed significantly in numerous aspects of their behaviours, males had greater overall activity than females, males spent more time locomoting and walking than females, males rested less than females, and males were less vigilant than females (figures 1*a* and 2*a*). This supports previous theories that males forgo rest and travel for longer periods of time to increase their chance at mating [10,26].

Some males travelled very long distances over the space of a day (Moimoi 10.4 km; Cayless, 9.4 km); overall, they did not locomote for significantly more distance or at faster speeds than females. Long travel distances have previously been recorded for breeding male northern quolls using GPS tracking as well as reported increases in home range size [26,54]. We can hypothesize two potential reasons for this. The first is that males will seek out areas with high density of females travelling large distances to find these locations and remain until the resource is exhausted. Locations with high density of females would benefit both male and female breeding strategies. For females, the increased scent marking of a densely populated area during a highly synchronized breeding rut would attract greater numbers of dominant males [51,55]. Similarly, males may use this strategy to increase their access to females. Male quolls seemed to intersperse days of increased movement, with days of lower movement. This was supported by the increased variance in mean distance/hour in males (*F*_265,517_ = 0.766, *p* = 0.014; figure 2*d*).

The second reason for the similarity in distance travelled could be that some males were captured post or nearing the end of their breeding season. If this was the case, male behaviour may be influenced by post-breeding senescence [20]. While the rut was synchronized in each locale, the different locations used for trapping seemed to have slightly different starts to the breeding rut. The timing of senescence is likely unique to individual males. Individual males showed very minimal movement during some days and a higher variance in distance travelled, which could represent senescence (figure 2*d*).

### 4.4. Restlessness in male northern quolls

The determinants of sleep (rapid eye movement, slow-wave sleep, etc.) were not measured in northern quolls; however, the percentage of inactivity (appropriate time when individuals could sleep) and the duration of inactive bouts (increased chance of experiencing REM or SWS) has been recorded. Inactivity has been used to determine resting time due to the limitations of measuring sleep determinants in wild roaming individuals; attempts have been made to record these sleep determinants using accelerometers with positive outcomes [56–58]. The increased activity and time spent locomoting, reduced frequency of resting and reduced duration of resting time in male northern quolls could explain their post-breeding die-off. While it is suggested in smaller dasyurids that the increase in corticosteroid is responsible for the die-off of males because of its effects on the immune and inflammatory systems, this was not reported for northern quolls [10]. The lack of sleep has not been explored in northern quolls, but the effects of sleep deprivation have been studied in rodent species [59–62].

Sleep-deprived rats experienced a decline in appearance despite engaging in daily grooming for more than an hour longer than control rats [60]. A similar decline in condition is reported in male northern quolls during their breeding season, which includes a notable increase in parasites which could be explained by a decrease in grooming efficiency [10]. The sleep-deprived rats also experienced significant weight loss compared to their control (17.9–21.6% reduction) despite increasing food intake by more than 80% compared to the original baseline and at least 60% more than the control rats [59,60,63]. This was attributed to an increased heart rate (approx. 10%), and energy expenditure (90%) calculated by calorie intake, food metabolized (80%) and weight loss [61]. Similar reductions in weight have also been reported in post-breeding male northern quolls [10]. Rats that were deprived of REM sleep were noticeably more aggressive in behaviour prompting changes to handling methods during this study [59]. Increased aggression has also been reported in male northern quolls; however, aggression in this instance might also be attributed to physiological changes during the breeding season [64].

The symptoms experienced by sleep-deprived rats have been recorded in post-breeding male northern quolls and could explain die-off that they experience [10,20,64]. Male northern quolls engaged in much less total resting behaviour than females (figure 1*a*). The 7.67% of resting behaviour in male northern quolls seems to be far less than the sleeping behaviour that is recorded in other marsupials (80% *D. marsupialis* and *Lutreolina crassicaudata*; 57% *Trichosurus vulpecula;* 48% *Potorous tridactylus*) [65]. However, due to the architecture of a sleep cycle, the duration of your sleeping bout is equally important. The duration of sleep cycles in common opossums, which are similar in trophic position and weight to northern quolls (important determinants of sleep cycle duration) [66], are reported to be 22.5 min (s.e. = 1.9) [35]. For male northern quolls, resting for more than 15 min is less frequent compared to female quolls, which suggests that males are getting less high-quality rest time in addition to an overall reduction in rest time (figures 1*a* and 2*c*).

From this data, it cannot be directly determined if male quolls did experience sleep deprivation; however, they do assume lying/resting behaviours less frequently and for shorter durations than females. Sleep deprivation, and associated symptoms, combined with the increased temporal activity, for a prolonged duration, would make recuperation impossible and could explain the causes of death recorded in the males after breeding season (e.g. they become easy prey, unable to avoid collisions, or die from exhaustion) [10]. Regardless, more research can be directed into understanding if breeding northern quolls do experience sleep deprivation during this season and if this is experienced by the wider Dasyuridae and Didelphidae families. Virginian opossums (*Didelphis virginiana*) undergo a similar physiological change to other semelparous species but do not experience the die-off, while Tasmanian devils (*Sacrophilus harrisii*) experience a similar loss in condition and a reduced immunocompetence [4,67]. If male northern quolls forgo sleep to the detriment of their survival, northern quolls become an excellent model species for the effects of sleep deprivation on body function.

## Data Availability

Data and code will be available at: https://figshare.com/s/9bc0bcff09d3d8a1082e. The data are provided in the electronic supplementary material [68].
